# Exergaming as Part of the Telerehabilitation Can Be Adequate to the Outpatient Training: Preliminary Findings of a Non-randomized Pilot Study in Parkinson's Disease

**DOI:** 10.3389/fneur.2021.625225

**Published:** 2021-03-16

**Authors:** Imre Cikajlo, Alma Hukić, Dejana Zajc

**Affiliations:** ^1^Research and Development Unit, University Rehabilitation Institute, Ljubljana, Slovenia; ^2^School of Engineering and Management, University of Nova Gorica, Nova Gorica, Slovenia

**Keywords:** perception, Parkinson's disease, exergaming, virtual reality, (tele)rehabilitation, object manipulation

## Abstract

Parkinson's disease is a long-term and progressive degenerative disorder of the nervous system, affecting primarily motor coordination, noticeable as a tremor in one hand. Recent studies reported on positive outcomes of intensive physiotherapy of upper extremities. We built a telerehabilitation system with virtual pick and place tasks for small scale hand movements, and designed a pilot study to find whether such exergaming as a telerehabilitation service provides comparable outcomes as an outpatient exergaming service. A non-randomized pilot trial was designed. Hospital outpatients (28/40) with Parkinson's disease were recruited. Those meeting the inclusion criteria were divided into two groups; seven outpatients were assigned to the home (H) group and 21 outpatients to the hospital (URI) group. Both groups received 10 days of exergaming over the course of 2 weeks, each daily session lasting a maximum of 1 h. Primary outcomes were clinical tests; Box and Blocks Test (BBT), Jebsen Hand Function Test (JHFT), and Unified Parkinson's Disease Rating Scale (UPDRS part III) were carried out before and after the study. Secondary outcomes were hand kinematics and exergaming results; number of successfully moved objects and task time. Statistical analysis was carried out to find significant (*p* < 0.05) differences and Cohen's U3 was used to determine effect sizes. The differences between the groups in gender (*p* = 0.781), age (*p* = 0.192), and duration of the disease (*p* = 0.195) were tested with Bartlett's test and no statistical differences were found with an F test. Both groups demonstrated statistically significant improvements in clinical test UDPRS III (*p* = 0.006 and *p* = 0.011) and the hospital group also in BBT (*p* = 0.002) and JHFT (*p* = 0.015) and with UDPRS III and JHFT even in favor of the home group (χ^2^ = 5.08, *p* = 0.024, χ^2^ = 7.76, *p* = 0.005). Nevertheless, the exergaming results show significant improvement after training (U3 > 0.86). Exergaming has already been suggested as an effective approach in the planning of rehabilitation tasks for persons with Parkinson's disease. We have prepared a pilot study demonstrating that exergaming at home with telerehabilitation support may provide comparable clinical outcomes. The study shall be followed by a randomized study with higher statistical power to provide clinical evidence. Nevertheless, carrying out even part of the rehabilitation program at home is crucial for the development of future telerehabilition clinical services.

**Clinical Trial Registration:** www.ClinicalTrials.gov, identifier: NCT03175107.

## Introduction

Parkinson's disease (PD) is a slowly progressing degenerative disease of the extrapyramidal system with an unknown cause. The disease often affects people in midlife, between 35 and 60 years of age, with men more likely to become ill than women (1). The main clinical signs of PD are muscle stiffness (rigidity), slowness of movement (bradykinesia), hand tremor, and postural disorders. The disease typically affects the patient's daily activities and thus, their quality of life at different ages. Currently, the degeneration of dopaminergic neurons that trigger changes in the basal ganglia network is treated with levodopa/dopamine (2). However, this may cause a decrease in responsiveness to the medication over time. At the same time, physiotherapy is becoming important in individual treatment of people with PD as they retain more than $\frac{3}{4}$ of all activities (3, 4). Balance, posture, and mobility related functions often impact the quality of life, as the functions of upper extremities are highly related to participation.

The voluntary activities of patients have proven to contribute to the functional improvement of movement, physical capacity, and other manual activities; balance, walking, reaching, grasping, etc. Exercise based computer games have been introduced to rehabilitation programs (5) and a promising approach seems to be rapidly developing. Several reports on functional progress and performance have been published, but an insufficient number of studies have been dedicated to safety and clinical benefits. Most of the studies have used commercial games for the world-wide public, and those were often found to be too complex (5). Some exceptions also considered safety and functional outcomes (balance, dynamic gait, and quality of life) with commercial outfits (Kinect Adventures^™^), reported in a feasibility study on positive outcomes (6). Positive effects of computerized cognitive training on several clinical outcomes were examined in older adults (7) and positive cognitive effects of video games have also been reported (8). Most of these studies have been dedicated to postural activities, balance (9), and a large range of motion movements using commercially available “exergames” and motion capture equipment (e.g., Kinect). In spite of the wide-use of commercial motion capture systems at home, the telerehabilitation of patients with neuromuscular diseases or disorders can present a safety issue. Particularly in a standing position, a standing frame is almost essential in (sub)acute stroke rehabilitation (10). Rehabilitation of upper extremities has been successfully implemented; no need for standing frame, no safety issues in the seated position (11, 12) in particular for telerehabilitation (13, 14). However, the majority of neuromuscular disorders may involve spasticity and may require additional passive or even active robotic equipment (e.g., Armeo^R^ Armotion^™^, Motore++, InMotion Wrist^™^, ArmAssist^R^, etc.), most of these are too complex, too expensive, or simply present a safety hazard for independent home use. (Tele)rehabilitation of the upper extremities in persons with PD rarely requires an active exoskeleton, but rather uses exergames (15). For persons with PD, accurate movements such as grasping and fine finger motions may present even more important tasks and may significantly contribute to the improvement of their quality of life, particularly when the medication plan remains unchanged. Such small range of motion tasks are feasible without a robotic device, and make use only of the tracking camera (16), gloves (17), or even electroencephalography (18).

Preliminary studies combining virtual reality technology with physiotherapy in persons with PD and older adults offer promising results (19, 20) in terms of feasibility, but were carried out in a supervised laboratory environment and thus do not provide a sufficient link between motor learning and clinical application. Therefore, we designed equipment specifically for clinical settings. Our goal based small virtual object manipulation task targets hand and finger dexterity in persons with PD (21). Furthermore, the technical solution has been implemented in the experimental telerehabilitation process. Before performing a large-scale study, we carried out an early non-randomized clinical study to check whether participants who had been discharged from hospital and continued treatment at home demonstrated similar clinical outcomes as outpatients without changing the medication plan.

## Materials and Methods

### Study Design and Regulation

**Figure 1:**
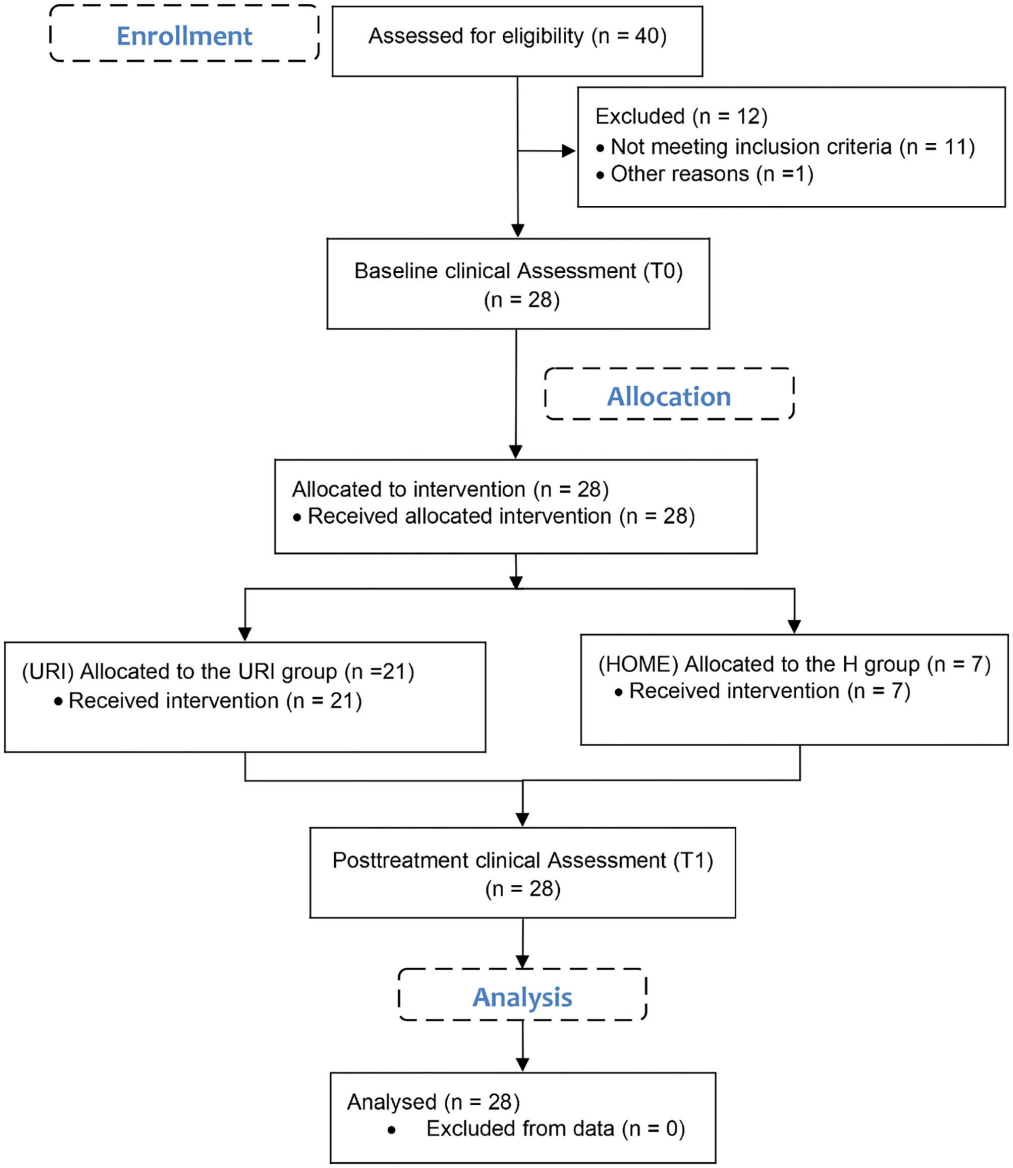
CONSORT flow diagram.

An experimental study with patients with PD was designed (Figure 1). Participants were included in the study according to the inclusion and exclusion criteria. Baseline clinical assessment was carried out on the day when all participants were still inpatients. On the same day, the patients were discharged from the hospital and allocated to two different groups by selection according to their location of residence and available technical equipment. Both the home (H) group and outpatient (URI) group participated in the post-treatment clinical assessment at the outpatient hospital. Primary outcome measures were clinical tests with questionnaire results as a supplement. Exergaming results were considered to be secondary outcome measures.

### (Tele)rehabilitation System

The telerehabilitation system was designed as a client-server model; a server and database running the front-end interface for a therapist working remotely, and client with the exergame, with data synchronization occurring after the accomplished session (16). The client software was running on the bare-bone computer (Intel NUC i7, Win10 OS) and was designed and programmed in the Unity3D (Unity Technologies, CA, USA) environment. The designed virtual environment (VE) consisted of a simulated grass floor, hidden reflecting walls, and a model of a treasure chest on the left for right-handed or on the right for the left-handed participants. We placed 10 virtual cubes of various colors in the VE, but the same physical model (weight, bounce stiffness, material, and size) to ensure repeatability of the process.

**Figure 2:**
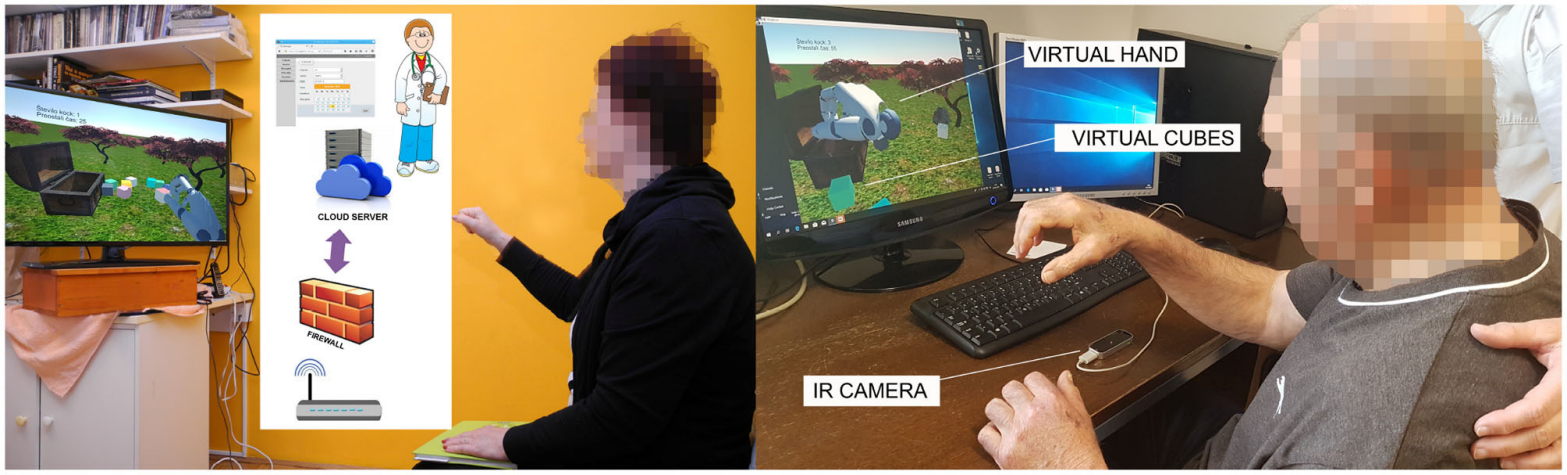
Participants at exergaming in home (left) and hospital (right) environment. Both groups used the same 10Cubes game and LMC. The telerehabilitation system for the home group recorded the data locally and transferred afterwards to the server.

The interaction object with the VE and the virtual object was a real hand-sized virtual model of the participant's hand (left or right). The participants could see the projection of their real hand and fingers as an avatar, a model of the metal hand. The kinematics of the virtual hand were adequately similar to the movement of the real hand and fingers. The movements of the real extremity were tracked by a small, mouse-sized infrared camera (Leap Motion Controller—LMC, Leap Motion Inc., CA, USA), that can detect 3D hand movement as well as the movements of fingers. The camera connects to the high-speed USB 3.0 port of the computer and requires a suitable graphic adapter (e.g., Nvidia GeForce series). The infrared camera requires light calibration or constant light conditions to operate properly with the pre-calibrated settings. The control software was written in C# using the LMC libraries within the MonoDevelop open-source environment (22). The assessed parameters were used to calculate the participant's performance. The recorded time and number of cubes placed successfully into the virtual chest were displayed to inform the participant about their performance. All parameters, including the hand kinematics, were simultaneously recorded on the local computer as an ASCII (^*^.txt) file and later sent to the remote server (Figure 2).

10Cubes game (Figure 2): The goal of the exergame was simple. Pick up and place 10 cubes, one by one into the open treasure chest within 2 min. One cube at a time should be picked up with a pincer grasp. If the participant completes the game before the time elapses, they receive a time bonus. If not, then the number of collected cubes counts as the final score.

### Participants

Twenty-eight persons (12 males and 16 females) with PD participated in the study. All participants were initially involved in the same rehabilitation program at the hospital. Inclusion criteria comprised: (a) Parkinson's disease or Parkinsonism with functional disorders in the upper extremities and minor problems with daily activities; (b) participants at level 2–3 according to the Hoehn and Yahr Scale (23). All tests were performed in the morning about 1–2 h after taking the medication to assure equal conditions for all participants. The recruited participants were divided into two groups according to their place of residence and the technical capabilities of their home:

- Seven patients in the home (H) group; three males and four females, 62.3 ± 7.3 years old, seven had the right side affected, with a duration of PD 5.8 ± 2.5 years.
- Twenty-one patients in the outpatient hospital (URI) group; nine males and 12 females, 69.5 ± 5.8 years old, three had the left side affected, 18 had the right side affected, and one had both sides affected, with a duration of PD 6.4 ± 4.5 years.

The study was approved by the local ethics committee and all participants provided a written consent for the publication of any potentially identifiable images or data included in this article.

### Research Protocol

The participants of the URI group were comfortably seated in front of the 22″ computer screen placed on a table. The LMC was placed on the desk in front of the participant (see Figure 2 right). Window blinds were used to assure appropriate constant lighting conditions. The participants of the H group were asked to sit comfortably in front of the 22″ monitor or 32″ TV screen and put the LMC on a small even surface above the subject's knees in order to cover the optimal working space (see Figure 2 left).

A skilled occupational therapist explained the goal of the study and the procedure of the task to each of the participants at the start of session zero (the trial session at the hospital). The therapist or the participant him(her)self-started the application, if computer skills allowed. The participants of the H group managed the application by themselves or were provided assistance by a relative or a caregiver.

The goal of the task was to pick up and place 10 small virtual cubes lying around the virtual environment into the open treasure chest by the more affected hand. If both or neither of the hands were affected, then the participant would use his/her dominant hand. For the purpose of the study, the model of virtual cubes used the same weight, material, and bouncing factor of the cubes and light conditions.

The participants of both groups were involved in the identical protocol. Differences in environment and equipment were unavoidable among the home participants. Each participant received 10 training therapies with the 10 Cubes exergame over the course of 2–3 weeks. Each session lasting ~30 min (max. 1 h) with breaks. Within the session, participants managed to accomplish the 10 Cubes task five times. Short breaks of 1–2 min between the trials were compulsory.

Before commencement of the sessions, the participants received a baseline clinical assessment. The clinical tests UPDRS motor function part (24), Jebsen Hand Function Test (25), and Box and Blocks Test (26) were carried out. All participants took the same tests at the post-treatment clinical assessment. All clinical tests were carried out by a skilled occupational therapist.

### Data Assessment

#### Clinical Outcomes

The clinical test Box and Blocks Test (26) is a low cost standardized rehabilitation measure that assesses unilateral gross manual dexterity. It is intended for evaluation of daily living activities, coordination, and dexterity of upper extremities. The participant moved blocks one by one from one compartment to another in 60 s. The outcome of the test was equal to the number of blocks, with more blocks indicating better function.

The Jebsen Hand Function Test (25) is a comprehensive rehabilitation measure test for uni-manual hand functions for the assessment of daily living activities. It comprises of seven sub-tests for dominant and non-dominant hands (writing a letter, card turning, picking up small objects, stacking checkers, stimulated feeding, moving light and heavy objects). The sub-tests measure speed, not the quality of movement. The outcome of each sub-test is the time taken to complete the task. Total score is the sum of times, with a lower score indicating better function.

The UPDRS (24) is a tool for general assessment of Parkinson's disease. We used only the motor function part III in the study in order to assess speech, facial expression, tremor at rest, action tremor of hands, rigidity, finger taps, hand movements, rapid alternating movements of hands, and actions related to balance and posture. Each point is graded from 0 (no impairment) to 4 (severe impairment). Therefore, a higher score indicates greater disability and zero points indicates the absence of disability.

After the study, the participants were asked to fill out the 39-Item Parkinson's Disease Questionnaire (PDQ-39) (27). However, we considered the results with precaution (28).

#### Exergaming Results

The kinematics of the hand were tracked by the LMC and analyzed online by computing the position of the cubes and determining the (un)successfully handled cubes, and monitoring the remaining time and offline analysis of the entire hand kinematic (22). The observed outcomes were the number of successfully picked and placed cubes and the remaining time.

### Sample Size Considerations

We set the confidence level to 95% in order to keep the average clinical value within expected limits. With an expected 10% margin of error and a population size of 40, we estimated that 29 participants would be required for the preliminary non-randomized study.

### Statistical Data Analysis

Differences between the H group and URI group in terms of gender, age, and duration of the disease were statistically checked for equal variances and normal distribution with Bartlett's Test and compared with an *F*-test (Matlab, MathWorks Inc., Natick MA, USA). Mean values and standard deviations were computed for the number of cubes and remaining time for each of the 10 sessions/days. Additionally, the statistical differences between the 1st and 10th sessions were tested with the Kruskal-Wallis Test, a non-parametric method. The significance level was set to *p* = 0.05. Matlab Statistical Toolbox (MathWorks Inc., Natick MA, USA) was used to manage and transform data, and to calculate the statistics. Effect sizes were determined with Cohen's U3 (29) analysis with the Measures of Effect Size (MES) Toolbox (30). The U3 defines the proportion of data from one group that were smaller than the median values of the other group. There was no effect at U3 = 0.5 and maximal at 1 when all group data at post-assessment were above the median of the data at baseline or 0 when all group data were below the median of the data at baseline (effect size: small 0.4/0.6, medium 0.3/0.7, and large 0.2/0.8).

Clinical tests were statistically examined separately for the H group and URI group. Data obtained in both groups were tested for normality and equality of variances with Bartlett's test. If the homogeneity of variances test did not fail (χ^2^, *p* < 0.05), we used the Student's *t*-test to compare the means of the clinical baseline and post-assessment. We hypothesized that clinical tests related to small object manipulation with hands or fingers can demonstrate statistically significant progress for both groups. Additionally, the effect sizes were examined by Cohen's U3. The statistical differences of the mean values between the H and URI groups were tested with the Kruskal-Wallis Test.

## Results

### Participants Characteristics

All 28 participants entering the study accomplished the 10 sessions according to the protocol and completed the assigned baseline and post-treatment clinical tests. The statistical test does not reject the null hypothesis that the variances in gender, age, and duration of the disease are equal across H and URI groups (Table 1).

**Table 1:** Analyzing differences between the home (H) group and hospital (URI) group.

**Variable**	**URI group**	**H group**	**Bartlett's test (χ^**2**^/p)**	***F*-test (*p*-value)**
Gender (M/F)	9/12	3/4	0.025/0.875	0.781
Age (mean/SD)	69.48 (5.78)	62.29 (7.32)	0.522/0.470	0.192
PD (years)	6.38 (4.48)	5.83 (2.48)	2.026/0.155	0.195

### Clinical Outcomes

**Figure 3:**
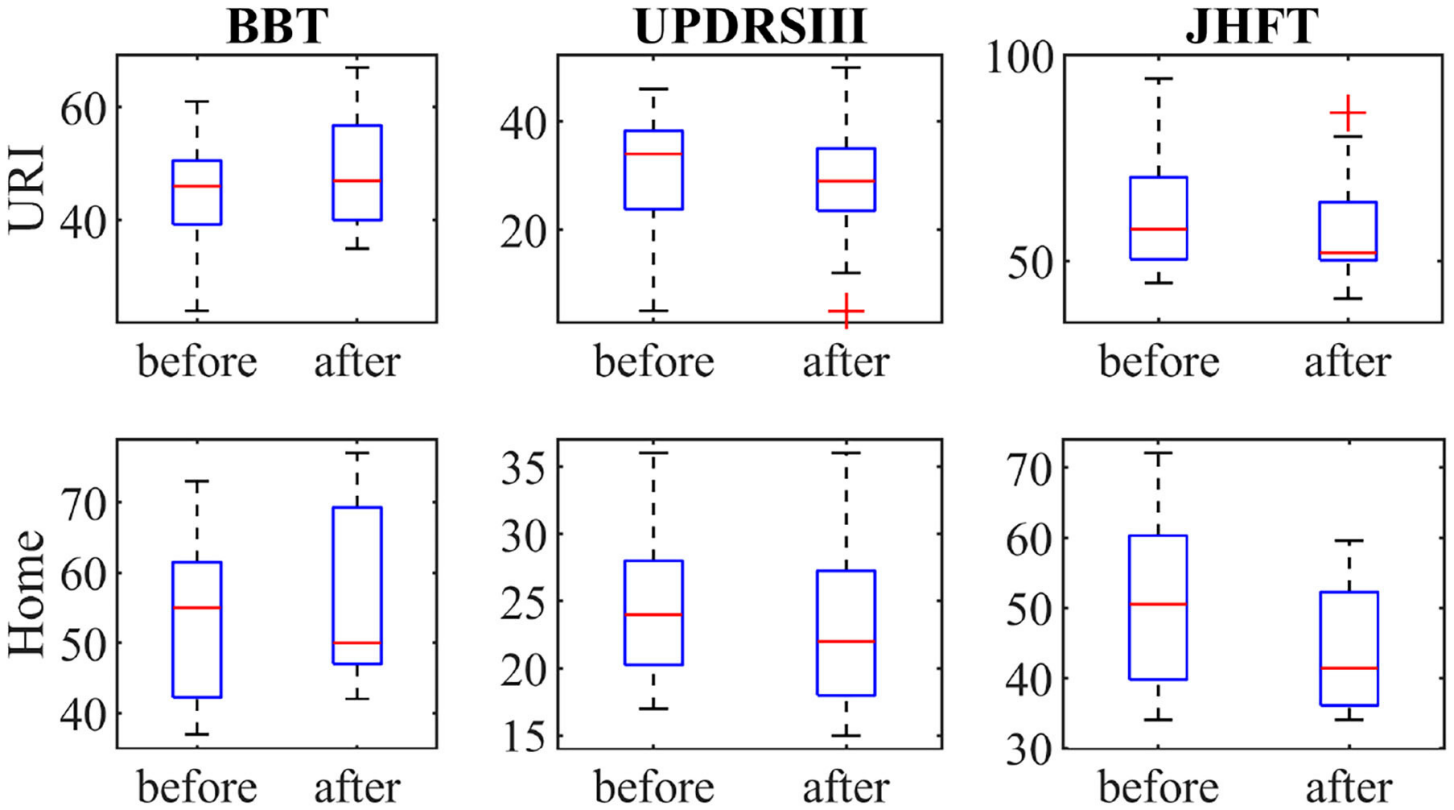
The outcomes of the clinical tests Box and Blocks Test (BBT), Unified Parkinson's disease rating scale (UPDRS) part III, and Jebsen Hand Function Test (JHFT) for the home and hospital (URI) groups before the commencement of the training and post-assessment.

The variances of the clinical tests at baseline and post-assessment were tested with Bartlett's test (χ^2^ < 1, *p* > 0.05) and the equal variance *t*-test was used to test the data. Significant improvements of function were found after the training with at least two clinical tests (Table 2). The URI group improved their score for all three clinical tests, BBT, UPDRS III, and JHFT, at post-assessment (Figure 3). The mean differences in the BBT and the UPDRS III were also statistically confirmed with *p* < 0.003 and *p* < 0.005, respectively. In the H group, improvements of function were found with all three tests, but changes were statistically significant for UPDRS III (*p* < 0.011) and JHFT (*p* < 0.015). The analysis of the effect sizes showed medium (U3 = 0.33) to large (U3 = 0.26) changes in JHFT, while changes in BBT scores were small in the H and URI groups (U3 = 0.43, U3 = 0.57, respectively) at post-assessment.

**Table 2:** Results of the clinical tests in both groups at baseline and post-treatment.

	**Hospital URI group**				**Home (H) group**				**Interaction**	
	**Baseline**		**Post**		**Baseline**		**Post**		**Kruskal-Wallis**	
**Test**	**Mean**	**SD**	**Mean**	**SD**	**Mean**	**SD**	**Mean**	**SD**	**χ^**2**^**	***p*-value**
BBT	44.6	9.3	49.1	9.8	52.9	12.9	56.6	13.6	3.38	0.066
UPDRS III	31.2	10.9	28.8	10.9	24.6	6.3	22.9	7.3	5.08	0.024^*^
JHFT	67.7	24.4	63.2	25.9	50.8	13.5	44.5	9.8	7.76	0.005^*^
	**Bartlett's**	***T*****-Test**	**Cohen's**		**Bartlett's**	***T*****-Test**	**Cohen'**			
	***χ***^**2**^**/*****p*****-value**	***p*****-value**	**U3**	**CI [x-y]**	***χ***^**2**^**/*****p*****-value**	***p*****-value**	**U3**	**CI [x-y]**		
BBT	0.052/0.820	0.003^*^	0.57	−7.37	0.017/0.896	0.204	0.42	−10.09		
UPDRS III	0.0001/0.997	0.005^*^	0.33	0.77	0.110/0.739	0.011^*^	0.43	0.55		
JHFT	0.066/0.797	0.068	0.26	−0.38	0.559/0.455	0.015^*^	0.29	1.70		

* *p < 0.05 statistical significant difference*.

The statistical differences in clinical scores between the H and URI groups were tested with the non-parametric Kruskal-Wallis Test for interactions due to unequal sample sizes. The JHFT and UPDRS III indicated significant differences (χ^2^ = 7.76, *p* = 0.005, χ^2^ = 5.08, *p* = 0.024, respectively) of means between the H group (mean 44.5 vs. 50.8 s, 22.9 vs. 24.6 s) and the URI group (mean 63.2 vs. 67.7 s, 28.8 vs. 31.2 s) as shown in Table 2.

**Figure 4:**
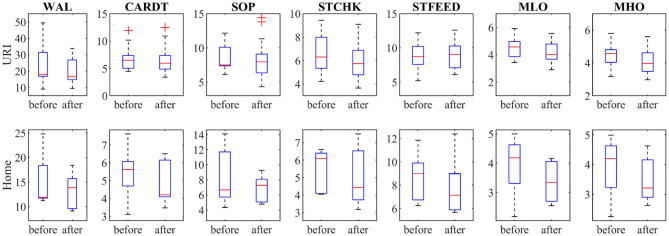
Shows the outcomes of the seven sub-tests of the Jebsen Hand Function Test for dominant/affected hand (WAL, writing a letter; CARDT, card turning; SOP, picking small objects; STCHK, stacking checkers; STFEED, stimulated feeding; MLO, moving light; MHO, moving heavy objects).

Figure 4 shows the outcomes of the seven sub-tests for the dominant/affected hand (writing a letter—WAL, card turning—CARDT, picking up small objects—SOP, stacking checkers—STCHK, stimulated feeding—STFEED, moving light objects—MLO and moving heavy objects—MHO). Both groups achieved lower scores (improvement) at post-assessment. Larger changes can be noticed for the H group, particularly in the sub-tests that require small object manipulation (MLO, WAL, SOP).

**Figure 5:**
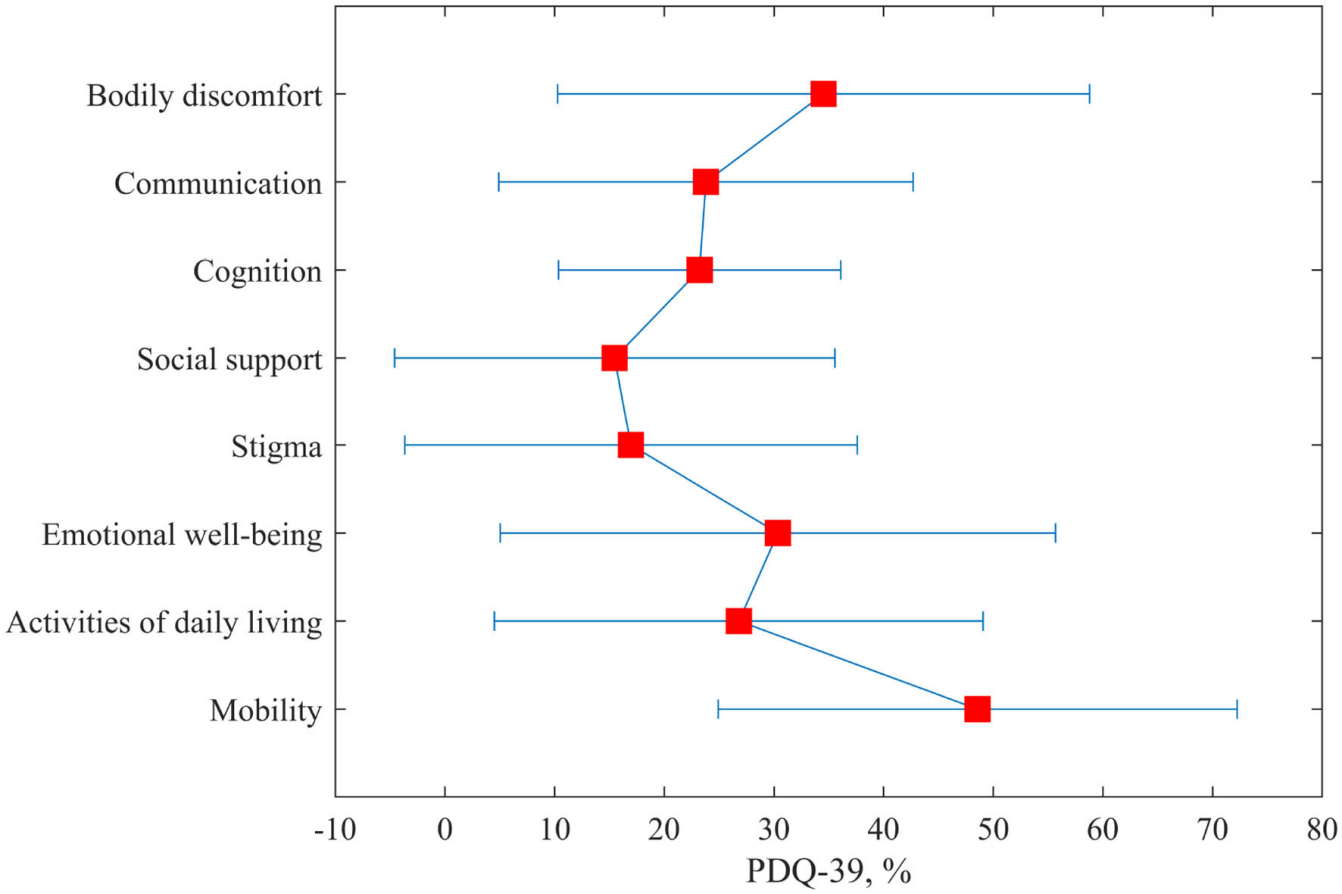
The outcomes of the Parkinson's Disease Questionnaire (PDQ-39) for the home group only.

The 39-Item Parkinson's Disease Questionnaire (PDQ-39) was filled by seven participants from the H group. The participants in general had severe difficulties with mobility, body discomfort, and also with emotional well-being (Figure 5). They did not feel stigmatized or lack of social support. The participants managed activities of daily living.

### Exergaming Results

**Figure 6:**
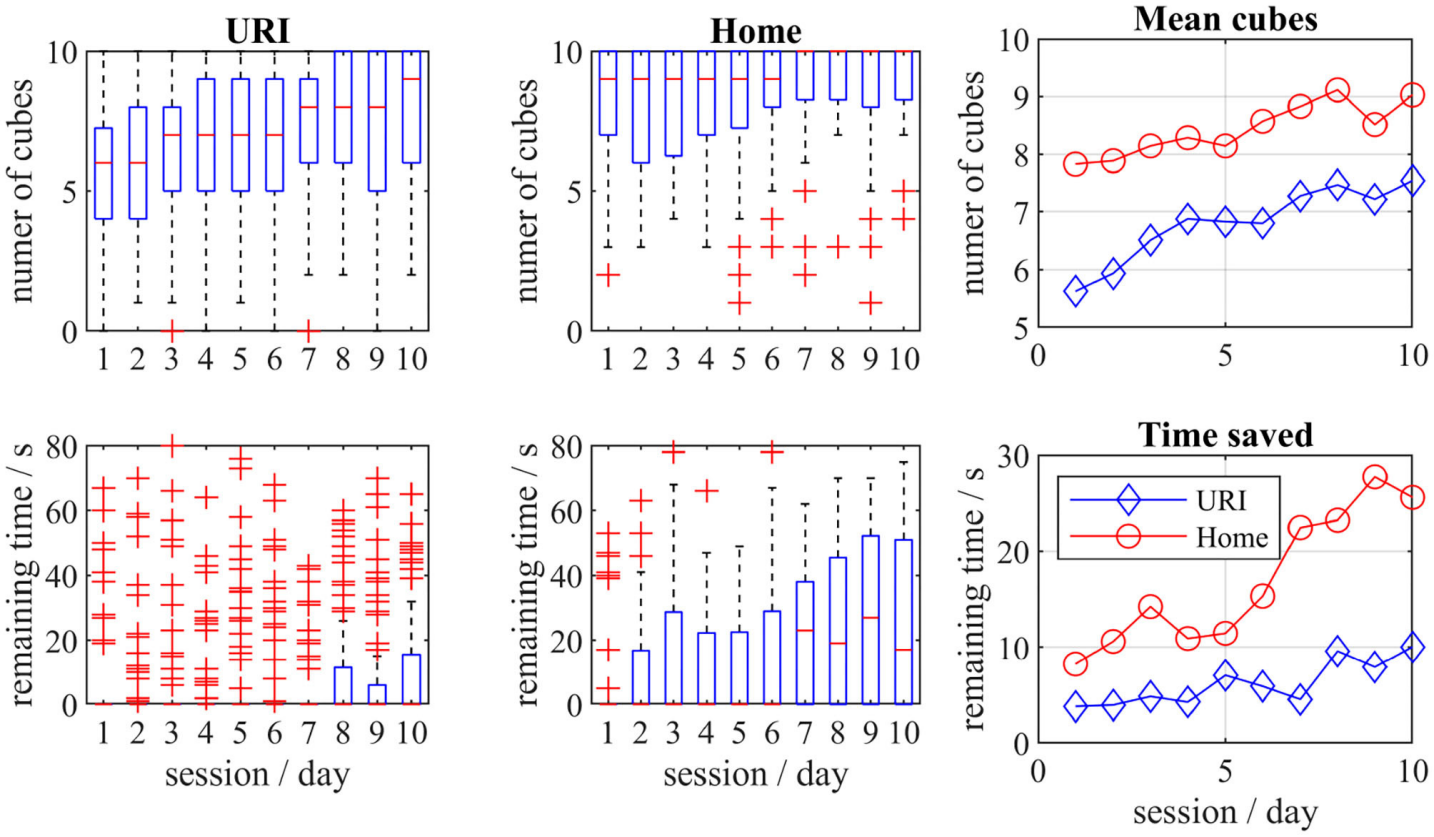
The boxplots for the number of successfully placed cubes and the remaining time for home and hospital (URI) groups. The mean values for both groups per sessions are also presented.

The participants of both groups have improved their exergame score, particularly the number of successfully placed cubes (Figure 6). The performance of the H group was also much faster, with more than 25 s remaining at the last session. All changes were substantially large (Cohen's U3 > 0.814). The URI group did not manage to save extra time (U3 = 0.5), however, some individuals in this group performed faster and saved up to 70 s (Figure 6).

The mean values between the H and URI groups were different for collected cubes as well as for the remaining time at post-assessment. The statistical differences between the groups were shown by the Kruskal-Wallis test (Table 3) for cubes (interaction, χ^2^ = 24.62, *p* = 6.98^*^10^−7^) and remaining time (interaction, χ^2^ = 11.33, *p* = 0.0008).

**Table 3:** Statistical differences between the hospital (URI) group and the home (H) group in exergaming.

**Variable**	**Cohen's U3**		**Kruskal-Wallis**	
	**URI group**	**H group**	**χ^**2**^**	***p*-value**
Number of cubes	0.867	0.857	24.62	0.00007^*^
Remaining time	0.500	0.814	11.33	0.0008^*^

*Additionally, the effect sizes (Cohen's U3) for baseline and post assessment is shown*.

* *p < 0.05 statistical significant difference*.

## Discussion

Safety, usability, and the patient's perception, particularly of telerehabilition, are major challenges of using novel technological solutions and methods in rehabilitation. The primary goal of telerehabilitation has always been the safety of participants. Technical safety is nowadays not questionable (Medical Device Regulation, 2020/561) (31), but rather acceptance, feasibility of the approach in remote or home environment, and clinical evidence. This may present a challenge in gait and balance training (32) but feasible and promising for the rehabilitation of upper extremities, particularly in the seated position. The patients improve the range of motion, grip muscle strength, coordination, movement velocity, and fine and gross dexterity. They can use non-immersive serious games or virtual reality and contactless measuring equipment (33). The gaming approach is often found to be motivating and challenging for patients, while game diversity and fun is of great importance (34). Fernández-González et al. reported on promising results using the LMC in patients with PD in the randomized control study. Significant improvements with clinical tests were observed despite the small number of participants. Our findings with a larger hospital group are in accordance with the reported results. The patients significantly improved their Box and Blocks Test score with dominant/more affected hand and UPDRS III scores using similar equipment with the virtual pick and place task. A substantial improvement (Cohen's U3 = 0.26) of JHFT outcomes was reported, but the statistical test showed marginal differences (*p* = 0.068). Insight into the sub-tests demonstrates progress with moving light objects, picking up small objects, and writing a letter. Further improvements in other sub-tasks would require additional exergames targeting different movements, cognition, and perception (35). The home group achieved a lower score in the moving light objects and picking up small objects sub-tasks and statistically significant improvement after the training (*p* = 0.015). The reason for better performance can be found in other sub-tests, i.e., writing a letter and stimulated feeding, the various tasks they usually do at home. The home group showed mean functional improvement of fine movements and gross manual dexterity with BBT, but statistically insignificant due to the high dispersion and small sample size.

Both groups of participants substantially improved their game scores and managed to save extra time. In fact, the home group was successful in gaming and their mean remaining time at the end of the trial was more than 25 s, meaning that the members of this group mastered the game. On the other hand, we have noticed that seven patients in the hospital were even better and two patients hardly managed to complete the game. This resulted in a great variation of results and may suggest that some patients have a more impaired pinching function than others. Also, the home group may have improved the response time of fingers to a stimulus quicker through the sessions (35). Even if we assume that the home group performs more physical reality tasks than the hospital group, Wang et al. demonstrated comparable outcomes with the virtual reality tasks (19). Visual motion stimuli contributed to the improvement of movement speed in persons with PD in the short-term. Recently, researchers (36) have demonstrated quantitatively positive results for upper extremities with immersive virtual tasks also using the LMC. Significant improvements of strength, fine and gross coordination, dexterity, and speed of movement were shown. The findings on comparable outcomes with the physical world lead to the validation of fully-immersive VR Box and Blocks Test (37). The authors suggested that virtual BBT could be used as a reliable indicator and may be accepted by clinicians and patients. However, the outcomes of our previous randomized control study show that there is no functional difference between immersive and non-immersive virtual tasks, except motivation (21). Motivation (38) could have an important impact on final results in both groups, home and hospital.

### Exergaming as Telerehabilitation Service

The LMC has previously been integrated into the home virtual rehabilitation system for stroke survivors (39). The system comprised simple goal oriented tasks for finger flexion/extension, wrist movement, and reaching with changing difficulty levels. The outcomes demonstrated improvement of upper extremity function and increased intrinsic motivation level. The participants maintained motivation for 12 weeks which could have an important impact on adherence and motor outcome. Motivation in chronic stroke can also be maintained by multi-user exergames (40). The outcomes of a study with Kinect^™^ (Microsoft, Inc., USA) suggested that the participants spent more time in multi-user training and achieved a higher Fugl-Meyer Assessment of Motor Recovery After Stroke Upper Extremity score. An interesting approach to involve active participation was the use of electroencephalography as neuro-feedback to control the interaction with the system (18). Such equipment can also be used for monitoring, despite the fact that the don and doff as well as the operation may present a technical issue for patients at home. Our study is focused on persons with PD who may not experience spastic movements and functional progress due to the progressive disease. However, the core of our telerehabilitation exergaming was in accordance with recent developments and studies; exergaming should be motivating, easy to use, and demonstrate comparable clinical effectiveness as the training in the hospital.

### Limitations

The system was designed as an easy-to-use, simple tool and does not require any special knowledge or technical skills. Despite simplification with a design devoid of an additional user interface for settings, it was not an easy task for the participants with PD to run the application. Data loss was prevented by saving them locally then uploading to the server afterwards.

We have noticed that successful collection of cubes before the elapsed time sweeten the pot. However, we are aware that the game score cannot be of relevant information for the clinician, but rather a good indicator of participant motivation/effort. We could also have applied the intrinsic motivation inventory to both groups (41).

The recruited number of patients of this mean age would have been enough for the estimated statistical power (0.8) with alpha set to 0.05. They could have been randomized into two groups, even 10 participants per group would have made the two independent sample study possible. Unfortunately, not all patients were eligible for the trial and we ended up with only seven who were capable of handling the technology. Although we found significant differences in outcomes across the groups with medium effect size, the small sample size in H group is a limiting factor. Consequently, there is a high risk of bias. A non-negligible factor would be the lack of motivation, ability to perform required exercises, poor sensibility or muscle tone.

Hereby, we suggest a randomized clinical trial, possibly multi-center to provide clinical evidence. Results can also be supported with the intrinsic motivation inventory to assess the patients' psychological behavior.

### Implications for Prospective Studies

The aim of this preliminary non-randomized study was to demonstrate that the location of supplemented occupational therapy is marginal and that the telerehabilitation approach may significantly change such therapy programs in the future. However, as the study did not provide sufficient clinical evidence, prospective randomized clinical trials in telerehabilitation settings are essential.

## Conclusion

The telerehabilitation system was initially designed for the proposed study; simple virtual reality task for small range of motion and precise manipulation, without specific user interface, short term evaluation protocol, and easy-to-use hardware and software. Indeed, small and precise movements are important and valuable for the daily life activities of persons with PD. Such activities increase participation and may potentially influence the progress of PD. The outcomes of the pilot study demonstrated comparable clinical outcomes indicating that part of the occupational therapy can be provided as telerehabilitation service. A limitation of such studies is often the small number of eligible participants. However, the COVID-19 pandemic and continuous pressure on rehabilitation centers have irrevocably altered the approach, the technology and its digital adoption for health care professionals and patients.

## Data Availability Statement

The original contributions presented in the study are included in the article/Supplementary Materials, further inquiries can be directed to the corresponding author/s.

## Ethics Statement

The study involving human participants was reviewed and approved by Ethics Committee of University Rehabilitation Institute, Republic of Slovenia (Approval Number: 13042015). The procedure was in accordance with the principles of the Declaration of Helsinki on biomedical research on human beings, the provisions of Council of Europe Convention on the Protection of Human Rights and Dignity of the Human Being with regard to the Application of Biology and Medicine (Oviedo Convention) and the principles of Slovenian Code of medical ethics. The patients/participants provided their written informed consent to participate in this study. Written informed consent was obtained from the individual(s) for the publication of any potentially identifiable images or data included in this article.

## Author Contributions

IC lead the research, made the analysis, and wrote the main structure of the manuscript. AH and DZ carried out the occupational therapy and clinical assessment, the participants selection, and coordination. All authors read and approved the final manuscript.

## Conflict of Interest

The authors declare that the research was conducted in the absence of any commercial or financial relationships that could be construed as a potential conflict of interest.

## References

[1] Wooten GF Currie LJ Bovbjerg VE Lee JK Patrie J. Are men at greater risk for Parkinson's disease than women? J Neurol Neurosurg Psychiatry. (2004) 75:637–9. 10.1136/jnnp.2003.020982 15026515 PMC1739032

[2] Blandini F Nappi G Tassorelli C Martignoni E. Functional changes of the basal ganglia circuitry in Parkinson's disease. Prog Neurobiol. (2000) 62:63–88. 10.1016/S0301-0082(99)00067-2 10821982

[3] Duncan RP Earhart GM. Measuring participation in individuals with Parkinson disease: relationships with disease severity, quality of life, and mobility. Disabil Rehabil. (2011) 33:1440–6. 10.3109/09638288.2010.533245 21091047

[4] Jankovic J. Parkinson's disease: clinical features and diagnosis. J Neurol Neurosurg Psychiatry. (2008) 79:368–76. 10.1136/jnnp.2007.131045 18344392

[5] Barry G Galna B Rochester L. The role of exergaming in Parkinson's disease rehabilitation: a systematic review of the evidence. J Neuroeng Rehabil. (2014) 11:33. 10.1186/1743-0003-11-33 24602325 PMC3984732

[6] Pompeu JE Arduini LA Botelho AR Fonseca MBF Pompeu SMAA Torriani-Pasin C. Feasibility, safety and outcomes of playing Kinect Adventures!^™^ for people with Parkinson's disease: a pilot study. Physiotherapy. (2014) 100:162–8. 10.1016/j.physio.2013.10.003 24703891

[7] Kueider AM Parisi JM Gross AL Rebok GW. Computerized cognitive training with older adults: a systematic review. PLoS ONE. (2012) 7:e40588. 10.1371/journal.pone.0040588 22792378 PMC3394709

[8] Torres ACS. Cognitive effects of video games on old people. Int J Disability Human Dev. (2011) 10:55–8. 10.1515/ijdhd.2011.003

[9] Yuan RY Chen SC Peng CW Lin YN Chang YT Lai CH. Effects of interactive video-game-based exercise on balance in older adults with mild-to-moderate Parkinson's disease. J Neuroeng Rehabil. (2020) 17:91. 10.1186/s12984-020-00725-y 32660512 PMC7359629

[10] Cikajlo I Rudolf M Goljar N Burger H Matjačić Z. Telerehabilitation using virtual reality task can improve balance in patients with stroke. Disabil Rehabil. (2012) 34:13–8. 10.3109/09638288.2011.583308 21864205

[11] Piron L Turolla A Agostini M Zucconi C Cortese F Zampolini M. Exercises for paretic upper limb after stroke: a combined virtual-reality and telemedicine approach. J Rehabil Med. (2009) 41:1016–102. 10.2340/16501977-0459 19841835

[12] Rogers JM Duckworth J Middleton S Steenbergen B Wilson PH. Elements virtual rehabilitation improves motor, cognitive, and functional outcomes in adult stroke: evidence from a randomized controlled pilot study. J Neuroeng Rehabil. (2019) 16:56. 10.1186/s12984-019-0531-y 31092252 PMC6518680

[13] McCue M Fairman A Pramuka M. Enhancing quality of life through telerehabilitation. Phys Med Rehabil Clin N Am. (2010) 21:195–205. 10.1016/j.pmr.2009.07.005 19951786

[14] Adams JL Myers TL Waddell EM Spear KL Schneider RB. Telemedicine: a valuable tool in neurodegenerative diseases. Curr Geriatr Rep. (2020) 9:72–81. 10.1007/s13670-020-00311-z 32509504 PMC7274219

[15] Gandolfi M Geroin C Dimitrova E Boldrini P Waldner A Bonadiman S. Virtual reality telerehabilitation for postural instability in Parkinson's disease: a multicenter, single-blind, randomized, controlled trial. Biomed Res Int. (2017) 2017:7962826. 10.1155/2017/7962826 29333454 PMC5733154

[16] Cikajlo I Hukić A Dolinšek I Zajc D Vesel M Krizmanič T. Can telerehabilitation games lead to functional improvement of upper extremities in individuals with Parkinson's disease? Int J Rehabil Res. (2018) 41:230–8. 10.1097/MRR.0000000000000291 29757774 PMC6092088

[17] Chiri A Cortese M de Almeida Riberio PR Cempini M Vitiello N Soekadar SR. A telerehabilitation system for hand functional training. In: Pons JL Torricelli D Pajaro M editors. Converging Clinical and Engineering Research on Neurorehabilitation. Berlin; Heidelberg: Springer-Verlag (2013). p. 1019–23. 10.1007/978-3-642-34546-3_167

[18] Navarro E González P López-Jaquero V Montero F Molina JP Romero-Ayuso D. Adaptive, multisensorial, physiological and social: the next generation of telerehabilitation systems. Front Neuroinform. (2018) 12:43. 10.3389/fninf.2018.00043 30042671 PMC6049338

[19] Wang CY Hwang WJ Fang JJ Sheu CF Leong IF Ma HI. Comparison of virtual reality versus physical reality on movement characteristics of persons with Parkinson's disease: effects of moving targets. Arch Phys Med Rehabil. (2011) 92:1238–45. 10.1016/j.apmr.2011.03.014 21718966

[20] Miller KJ Adair BS Pearce AJ Said CM Ozanne E Morris MM. Effectiveness and feasibility of virtual reality and gaming system use at home by older adults for enabling physical activity to improve health-related domains: a systematic review. Age Ageing. (2014) 43:188–95. 10.1093/ageing/aft194 24351549

[21] Cikajlo I Peterlin Potisk K. Advantages of using 3D virtual reality based training in persons with Parkinson's disease: a parallel study. J Neuroeng Rehabil. (2019) 16:119. 10.1186/s12984-019-0601-1 31623622 PMC6798369

[22] Cikajlo I Pogačnik M. Movement analysis of pick-and-place virtual reality exergaming in patients with Parkinson's disease. Technol Health Care. (2020) 28:391–402. 10.3233/THC-191700 32200361

[23] Goetz CG Poewe W Rascol O Sampaio C Stebbins GT Counsell C. Movement Disorder Society Task Force report on the Hoehn and Yahr staging scale: status and recommendations the Movement Disorder Society Task Force on rating scales for Parkinson's disease. Mov Disord. (2004) 19:1020–8. 10.1002/mds.20213 15372591

[24] Movement Disorder Society Task Force on Rating Scales for Parkinson's Disease. The Unified Parkinson's Disease Rating Scale (UPDRS): status and recommendations. Mov Disord. (2003) 18:738–50. 10.1002/mds.10473 12815652

[25] Mak MKY Lau ETL Tam VWK Woo CWY Yuen SKY. Use of Jebsen Taylor Hand Function Test in evaluating the hand dexterity in people with Parkinson's disease. J Hand Ther. (2015) 28:389–95. 10.1016/j.jht.2015.05.002 26227308

[26] Kontson K Marcus I Myklebust B Civillico E. Targeted box and blocks test: normative data and comparison to standard tests. PLoS ONE. (2017) 12:e0177965. 10.1371/journal.pone.0177965 28542374 PMC5438168

[27] Jenkinson C Fitzpatrick R Peto V Greenhall R Hyman N. The Parkinson's Disease Questionnaire (PDQ-39): development and validation of a Parkinson's disease summary index score. Age Ageing. (1997) 26:353–7. 9351479 10.1093/ageing/26.5.353

[28] Hagell P Nilsson MH. The 39-Item Parkinson's Disease Questionnaire (PDQ-39): is it a unidimensional construct? Ther Adv Neurol Disord. (2009) 2:205–14. 10.1177/1756285609103726 21179529 PMC3002633

[29] Cohen J. Statistical Power Analysis for the Behavioral Sciences. Hillsdale, NJ: L. Erlbaum Associates (1988).

[30] Hentschke H Stüttgen MC. Computation of measures of effect size for neuroscience data sets. Eur J Neurosci. (2011) 34:1887–94. 10.1111/j.1460-9568.2011.07902.x 22082031

[31] EUR-Lex. EUR-Lex - L:2017:117:TOC – EN. (2017). Available at: https://eur-lex.europa.eu/legal-content/EN/TXT/?uri=OJ:L:2017:117:TOC (accessed October 30, 2020).

[32] Held JP Ferrer B Mainetti R Steblin A Hertler B Moreno-Conde A. Autonomous rehabilitation at stroke patients home for balance and gait: safety, usability and compliance of a virtual reality system. Eur J Phys Rehabil Med. (2018) 54:545–53. 10.23736/S1973-9087.17.04802-X 28949120

[33] Fernández-González P Carratalá-Tejada M Monge-Pereira E Collado-Vázquez S Sánchez-Herrera Baeza P Cuesta-Gómez A. Leap motion controlled video game-based therapy for upper limb rehabilitation in patients with Parkinson's disease: a feasibility study. J Neuroeng Rehabil. (2019) 16. 10.1186/s12984-019-0593-x 31694653 PMC6836460

[34] Hung Y-X Huang P-C Chen K-T Chu W-C. What do stroke patients look for in game-based rehabilitation: a survey study. Medicine. (2016) 95:e3032. 10.1097/MD.0000000000003032 26986120 PMC4839901

[35] Oña ED Balaguer C Cano-De La Cuerda R Collado-Vázquez S Jardón A. Effectiveness of serious games for leap motion on the functionality of the upper limb in Parkinson's disease: a feasibility study. Comput Intell Neurosci. (2018) 2018:7148427. 10.1155/2018/7148427 29849550 PMC5925003

[36] Sánchez-Herrera-Baeza P Cano-De-la-Cuerda R Oña-Simbaña ED Palacios-Ceña D Pérez-Corrales J Cuenca-Zaldivar JN. The impact of a novel immersive virtual reality technology associated with serious games in parkinson's disease patients on upper limb rehabilitation: a mixed methods intervention study. Sensors. (2020) 20:2168. 10.3390/s20082168 32290517 PMC7218715

[37] Oña ED Jardón A Cuesta-Gómez A Sánchez-Herrera-baeza P Cano-De-la-cuerda R Balaguer C. Validity of a fully-immersive VR-based version of the box and blocks test for upper limb function assessment in Parkinson's disease. Sensors. (2020) 20:2773. 10.3390/s20102773 32414177 PMC7285781

[38] Goršič M Cikajlo I Novak D. Motivation and exercise intensity in competition and cooperation between a patient and unimpaired person in arm rehabilitation. In: Proceedings of the 2016 International Conference on NeuroRehabilitation. Segovia (2016).

[39] Fluet GG Qiu Q Patel J Cronce A Merians AS Adamovich SV. Autonomous use of the home virtual rehabilitation system: a feasibility and pilot study. Games Health J. (2019) 8:432–8. 10.1089/g4h.2019.0012 31769724 PMC7133442

[40] Thielbar KO Triandafilou KM Barry AJ Yuan N Nishimoto A Johnson J. Home-based upper extremity stroke therapy using a multiuser virtual reality environment: a randomized trial. Arch Phys Med Rehabil. (2020) 101:196–203. 10.1016/j.apmr.2019.10.182 31715140

[41] Goršič M Cikajlo I Novak D. Competitive and cooperative arm rehabilitation games played by a patient and unimpaired person: effects on motivation and exercise intensity. J Neuroeng Rehabil. (2017) 14:23. 10.1186/s12984-017-0231-4 28330504 PMC5363008

